# Interferon gamma-related gene signature based on anti-tumor immunity predicts glioma patient prognosis

**DOI:** 10.3389/fgene.2022.1053263

**Published:** 2023-01-13

**Authors:** Zhe Zhang, Xiaoli Shen, Zilong Tan, Yuran Mei, Tianzhu Lu, Yulong Ji, Sida Cheng, Yu Xu, Zekun Wang, Xinxian Liu, Wei He, Zhen Chen, Shuhui Chen, Qiaoli Lv

**Affiliations:** ^1^ Department of Neurosurgery, The Second Affiliated Hospital of Nanchang University, Nanchang, Jiangxi, China; ^2^ Jiangxi Key Laboratory of Translational Cancer Research, Jiangxi Cancer Hospital, Nanchang, Jiangxi, China; ^3^ Department of Radiation Oncology and Head and Neck Surgery, Jiangxi Cancer Hospital, Nanchang, Jiangxi, China; ^4^ Department of Radiation Oncology, Jiangxi Cancer Hospital of Nanchang University, Nanchang, Jiangxi, China

**Keywords:** interferon gamma, glioma, tumor microenvironment, immune signature, prognosis

## Abstract

**Background:** Glioma is the most common primary tumor of the central nervous system. The conventional glioma treatment strategies include surgical excision and chemo- and radiation-therapy. Interferon Gamma (IFN-γ) is a soluble dimer cytokine involved in immune escape of gliomas. In this study, we sought to identify IFN-γ-related genes to construct a glioma prognostic model to guide its clinical treatment.

**Methods:** RNA sequences and clinicopathological data were downloaded from The Cancer Genome Atlas (TCGA) and the China Glioma Genome Atlas (CGGA). Using univariate Cox analysis and the Least Absolute Shrinkage and Selection Operator (LASSO) regression algorithm, IFN-γ-related prognostic genes were selected to construct a risk scoring model, and analyze its correlation with the clinical features. A high-precision nomogram was drawn to predict prognosis, and its performance was evaluated using calibration curve. Finally, immune cell infiltration and immune checkpoint molecule expression were analyzed to explore the tumor microenvironment characteristics associated with the risk scoring model.

**Results:** Four out of 198 IFN-γ-related genes were selected to construct a risk score model with good predictive performance. The expression of four IFN-γ-related genes in glioma tissues was significantly increased compared to normal brain tissue (*p* < 0.001). Based on ROC analysis, the risk score model accurately predicted the overall survival rate of glioma patients at 1 year (AUC: The Cancer Genome Atlas 0.89, CGGA 0.59), 3 years (AUC: TCGA 0.89, CGGA 0.68), and 5 years (AUC: TCGA 0.88, CGGA 0.70). Kaplan-Meier analysis showed that the overall survival rate of the high-risk group was significantly lower than that of the low-risk group (*p* < 0.0001). Moreover, high-risk scores were associated with wild-type *IDH1*, wild-type *ATRX*, and 1P/19Q non-co-deletion. The nomogram predicted the survival rate of glioma patients based on the risk score and multiple clinicopathological factors such as age, sex, pathological grade, and *IDH* Status, among others. Risk score and infiltrating immune cells including CD8 T-cell, resting CD4 memory T-cell, regulatory T-cell (Tregs), M2 macrophages, resting NK cells, activated mast cells, and neutrophils were positively correlated (*p* < 0.05). In addition, risk scores closely associated with expression of immune checkpoint molecules such as PD-1, PD-L1, CTLA-4, LAG-3, TIM-3, TIGIT, CD48, CD226, and CD96.

**Conclusion:** Our risk score model reveals that IFN-γ -associated genes are an independent prognostic factor for predicting overall survival in glioma, which is closely associated with immune cell infiltration and immune checkpoint molecule expression. This model will be helpful in predicting the effectiveness of immunotherapy and survival rate in patients with glioma.

## Introduction

Glioma is the most common primary tumor of the central nervous system, accounting for approximately 75% malignant primary brain tumors in adults (Ostrom et al., 2017). Gliomas usually originate from glial cells or other progenitor cells and are accordingly termed as astrocytoma, oligodendroglioma, oligodendroglioma, and ependymoma (Zhang et al., 2012). According to the new classification of central nervous system tumors by the World Health Organization (WHO) in 2016, gliomas can be classified into grade I to IV (Villa et al., 2018). Higher grade gliomas (grade IV) are the most lethal glioma (also called as glioblastoma, GBM) that have poor prognosis, with a median OS of only 15 months (Bleeker et al., 2012). Conventional treatment for gliomas includes surgical resection combined with radiation and/or chemotherapy. Although immunotherapy, targeted therapy, and combination therapy have been developed, the immune regulation and immune escape mechanisms used by glioblastoma pose considerable challenges to immunotherapy (Gieryng et al., 2017).

Interferons are highly species-specific glycoproteins that exert antiviral, anti-proliferative, anti-tumor, and immunoregulatory effects, and play pivotal role in coordinating immune response (Gresser, 1990). IFN-γ is a member of the type II IFN family. The mouse and human IFN-γ proteins are encoded by a 6 kb gene consisting of four exons and three introns located on exons 10 and 12, respectively (Trent et al., 1982). IFN-γ protein is a homodimer formed by non-covalent binding of two 17 kDa polypeptide subunits (Ealick et al., 1991). IFN-γ is secreted primarily by lymphocytes (CD4^+^ T helper type 1 (Th1) cells and CD8^+^ cytotoxic T-cell) (Kasahara et al., 1983; Corthay et al., 2005), gamma delta T-cell (Gao et al., 2003), and natural killer (NK) cells (Keppel et al., 2015) and plays an important role in coordinating innate and adaptive immune responses against viruses, bacteria, and tumors. IFN-γ can also promote pathological inflammatory process (Ni and Lu, 2018), and its involvement is positively associated with survival in cancer patients. Therefore, it is necessary to study the immunoregulatory effects of IFN-γ in tumor microenvironment (TME) (Castro et al., 2018).

In this study, we analyzed gene expression and clinical data of glioma samples obtained from The Cancer Genome Atlas (TCGA) database. Next, a risk score model based on IFN-γ genes was constructed using minimum absolute contraction and selection operator (LASSO) regression analysis and Cox regression analysis and validated in the Chinese Glioma Genome Atlas (CGGA) dataset. The potential relationship between the risk scoring model and clinicopathological features was analyzed using a nomogram. In addition, we analyzed the risk scoring model in predicting glioma prognosis based on immune status of TME, its relationship with immune checkpoint molecule expression, and its potential role in predicting immunotherapy outcomes. Considering the close correlation between IFN-γ and clinical treatment outcomes, we believe that our predictive model will be a useful reference for the future research studies in this field.

## Materials and methods

### Data collection

We collected transcriptome data and clinical prognosis information of 689 glioma patients from TCGA portal (https://portal.gdc.cancer.gov/); data of 212 normal patients were used as control. Only samples for which information related to complete time of life, status of life, and clinicopathological type including patient age, gender, glioma grade, *IDH* state, and pathological type were available, included in analysis. In addition, transcriptome data and clinical data of 367 glioma patients were downloaded from the CGGA portal (http://www.cgga.org.cn/) (Liu et al., 2018) for verifying results. We collected 30 primary glioma samples and 10 normal brain tissue samples from the Jiangxi Cancer Hospital (2020ky074).

### Gene set enrichment analysis

GSEA is performed to determine whether a set of pre-defined genes shows statistically significant and consistent differences between two biological states. Data of glioma and normal samples were subjected to GSEA (Subramanian et al., 2005) (https://www.gsea-msigdb.org/gsea/index.jsp). Significant differences in GSEA were verified by normalized enrichment score (NES) and error detection rate (FDR). Furthermore, we subjected our data to the Annotation, the Visualization, and Integrated the Discovery (DAVID) (Huang et al., 2009) (https://david.ncifcrf.gov/), Gene ontology (GO), and Kyoto Encyclopedia of Genes and Genomes (KEGG) pathway analyses. The pathway enrichment criteria were *p* < 0.05 and FDR< 0.05.

### Construction and validation of IFN-γ -related gene signature

First, we used “survival” and “glmnet” R software packages (Friedman et al., 2010) and performed univariate Cox regression analysis and the LASSO regression analysis to screen survival-related genes in glioma patients. Multivariate Cox regression analysis was performed to screen genes that could be used as independent prognostic factors for OS, and their regression coefficients were calculated. The risk score for each glioma patient was calculated as follows: Risk score = [Expression of Gene 1× coefficient]+[Expression of Gene 2× Coefficient]++... [Expression of Gene n× coefficient] Patients were further divided into high - and low-risk groups based on the median risk score. Kaplan-Meier method was used to compare survival differences between high- and low-risk groups. Finally, the “survival ROC” R software package (Heagerty et al., 2000) was used to establish time-dependent receiver operating characteristic (ROC) curve analysis (including 1-, 3-, and 5-year survival rate) to evaluate the sensitivity and accuracy of risk score. In addition, we used CGGA data set to verify the risk scoring model and generate Kaplan-Meier survival curve and survival ROC curve.

### RNA extraction and quantitative real-time PCR (RT-qPCR)

RNA was extracted from tissues using TRIzol reagent (TaKaRa, Shiga, Japan). cDNA synthesis was performed using PrimeScript RT kit (RR047A, TaKaRa). Real-time quantitative PCR was performed using a standard SYBR Green PCR kit (Takara, RR820A). We used the 2^−ΔΔCT^ method for calculations. The primers for the mRNA TNFAIP6 were 5′-TGC​TAC​AAC​CCA​CAC​GCA​AA-3' (forward) and 5′-CTC​AGG​TGA​ATA​CGC​TGA​CCA-3' (reverse). The primers for the mRNA PSMB2 were 5′- ATC​CTC​GAC​CGA​TAC​TAC​ACA​C-3' (forward) and 5′-GAA​CAC​TGA​AGG​TTG​GCA​GAT -3" (reverse). The primers for mRNA IRF4 were 5′-GCG​GTG​CGC​TTT​GAA​CAA​G-3' (forward) and 5′- ACA​CTT​TGT​ACG​GGT​CTG​AGA-3' (reverse). The primers for mRNA IFNAR2 were 5′-TCA​TGG​TGT​ATA​TCA​GCC​TCG​T-3' (forward) and 5′-AGT​TGG​TAC​AAT​GGA​GTG​GTT​TT -3" (reverse). The primers for GAPDH, 5′-CCC​ATC​ACC​ATC​TTC​CAG​GAG-3' (forward) and 5′-GTT​GTC​ATG​GAT​GAC​CTT​GGC-3' (reverse).

### Analysis of infiltrating immune cells

To investigate the correlation between the risk model based on IFN-γ associated genes and TME, we used ESTIMATE R package (Yoshihara et al., 2013) and CIBERSORT (https://cibersort.stanford.edu/) (Newman et al., 2015) to determine the TME score and the proportion of 22 kinds of infiltrating immune cells. Furthermore, we applied the Wilcox test to compare the differentially infiltrating immune cells between the high-rated and low-rated groups.

### Construction of prognostic nomogram

A nomogram can be used to combine multiple variables to diagnose or predict the probability of disease onset or progression. Using the “rms” R software package and the prognostic and clinicopathological features of the IFN-γ -associated gene risk score model, we developed a nomogram to predict the prognosis of glioma patients. Simultaneously, calibration plots were generated to compare the predicted values with actual survival rates to evaluate the accuracy of the nomogram.

### Statistical analysis

Kaplan-Meier method was used for survival analysis, and log-rank test was used to evaluate OS differences between groups. Univariate and multivariate analyses were performed using Cox proportional risk model to determine whether the risk scoring model could accurately predict prognosis of glioma patients. The violin diagram was drawn using the “violot” R software package. In addition, we performed an independent *t*-test to assess the relationship between IFN-γ -associated genes and various clinicopathological factors. SPSS (Version 26.0) and R software (Version 4.1.0) were used for all statistical analysis and generating charts. Results with *p* < 0.05 were considered statistically significant.

## Results

### Identification of IFN-γ related genes

**FIGURE 1:**
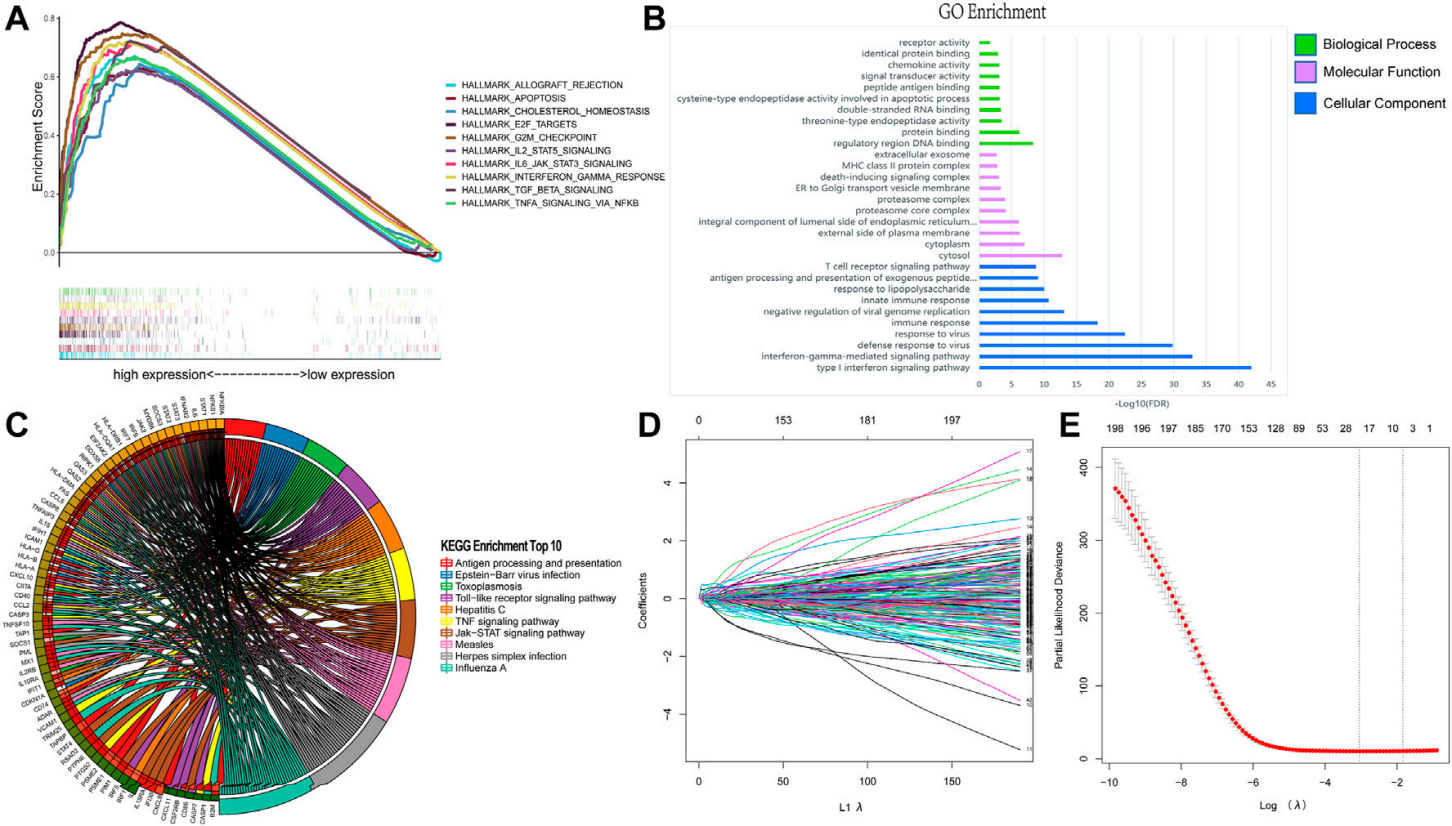
Identification of IFN-γ related genes. **(A)** GSEA analysis of glioma and normal samples from TCGA database. Enrichment analysis of 198 IFN-γ - related genes: **(B)** Enriched GO terms. **(C)** Kyoto Encyclopedia of Genes and Genomes Pathway. **(D, E)** Establishment and evaluation of risk scoring model based on IFN-γ-associated genes.

By performing GSEA of glioma and normal samples, specific gene sets were obtained. Twenty-six gene sets related to complement system, inflammatory response, interferon gamma response, mitotic spindle, Kras signaling, E2f targets, allograft rejection, IL2/Stat5 signaling, mTORC1 signaling, and MYC target v1, among others were enriched. (Figure 1A; Table 1). We selected 198 genes (*p* = 0.029) to further analyze the relationship between the function of the IFN-γ response-related genes and glioma patients prognosis. Functional enrichment analysis showed that the signaling pathway associated with the IFN-γ related gene was “Influenza A" (Figure 1C). Moreover, GO analysis revealed that in biological process (BP), molecular function (MF), and cellular component (CC), the gene was mainly involved in “immune response” and “regulatory region” among others. (Figure 1B).

**TABLE 1:** Gene sets enriched in normal and glioma patients.

GS follow link to MSigDB	Size	Es	NOM p-val	Rank at Max
COMPLEMENT	200	0.598396	0.03198294	12,533
INFLAMMATORY RESPONSE	199	0.631354	0.04535637	11,059
INTERFERON GAMMA RESPONSE	198	0.720191	0.02941177	10,281
MITOTIC SPINDLE	198	0.632598	0.04670913	14,770
KRAS SIGNALING UP	196	0.594534	0.0231579	10,234
E2F TARGETS	195	0.786049	0	9399
ALLOGRAFT REJECTION	195	0.670881	0.02547771	11,552
IL2 STAT5 SIGNALING	195	0.622643	0.02708333	12,435
MTORC1 SIGNALING	195	0.647061	0.0443038	14,672
MYC TARGETS V1	194	0.723588	0.0131291	13,140
G2M CHECKPOINT	190	0.747303	0.00408998	9630
APOPTOSIS	159	0.630793	0.02291667	12,308
DNA REPAIR	147	0.686525	0.02141328	12,039
INTERFERON ALPHA RESPONSE	95	0.745016	0.04661017	11,450
PROTEIN SECRETION	95	0.673518	0.02869757	10,966
IL6 JAK STAT3 SIGNALING	87	0.718659	0.02736842	10,998
CHOLESTEROL HOMEOSTASIS	73	0.64373	0.00632911	12,308
MYC TARGETS V2	58	0.706929	0.04814005	14,364
TGF BETA SIGNALING	54	0.722212	0.00430108	10,914
WNT BETA CATENIN SIGNALING	42	0.650237	0.02620087	6137
NOTCH SIGNALING	32	0.693124	0.00652174	6518

### Establishment and evaluation of risk scoring model based on IFN-γ-associated genes

**FIGURE 2:**
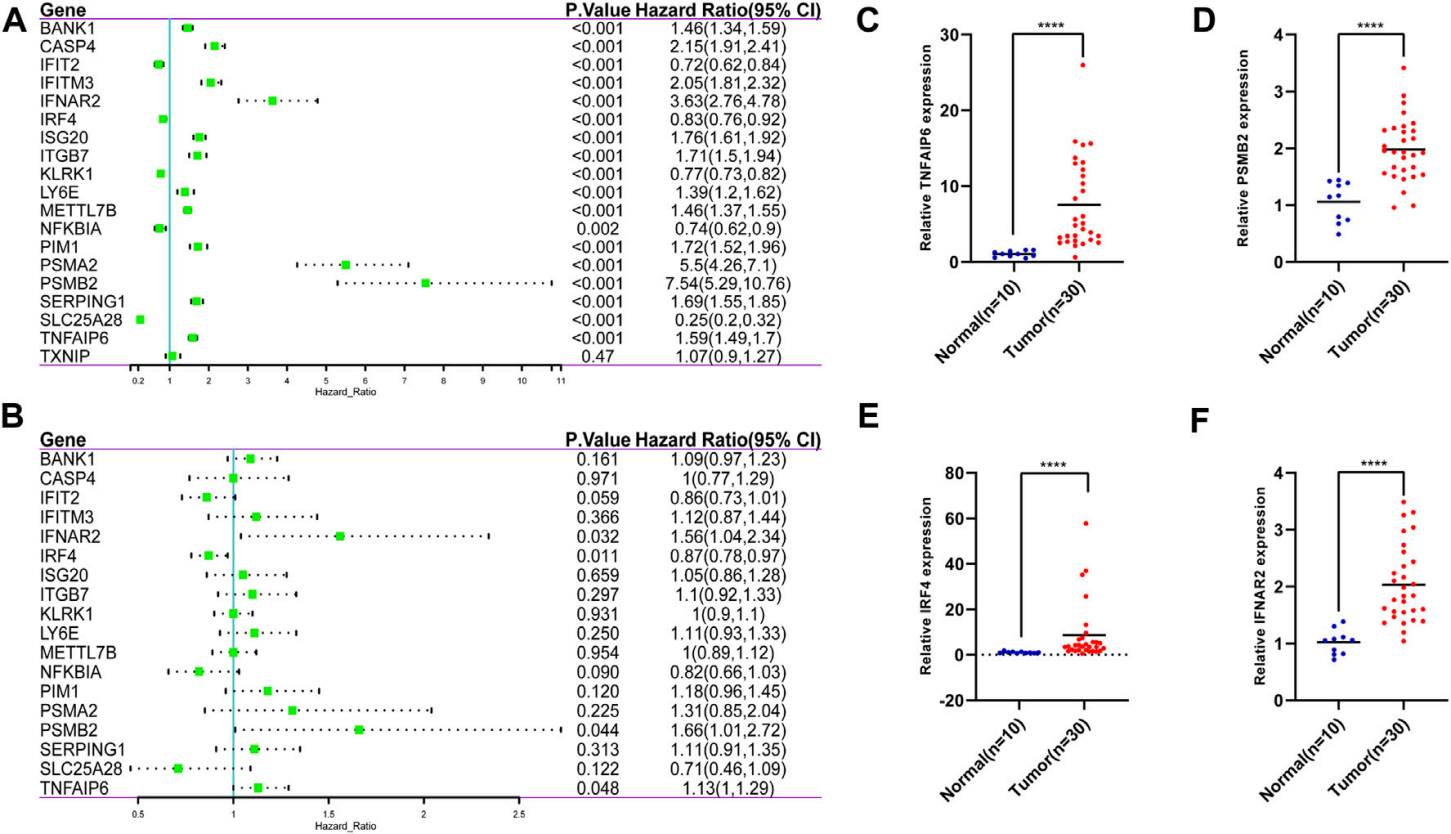
19 IFN-γ -associated genes were selected by the LASSO regression analysis. **(A)** 18 IFN-γ - associated genes were selected by univariate Cox analysis. **(B)** 4 IFN-γ - associated genes were selected by multivariate Cox analysis. The expressions of TNFAIP6 **(C)**, PSMB2 **(D)**, IRF4 **(E)** and IFNAR2 **(F)** were significantly upregulated in glioma tissues compared to normal brain tissues. ****p < 0 .0001.

**FIGURE 3:**
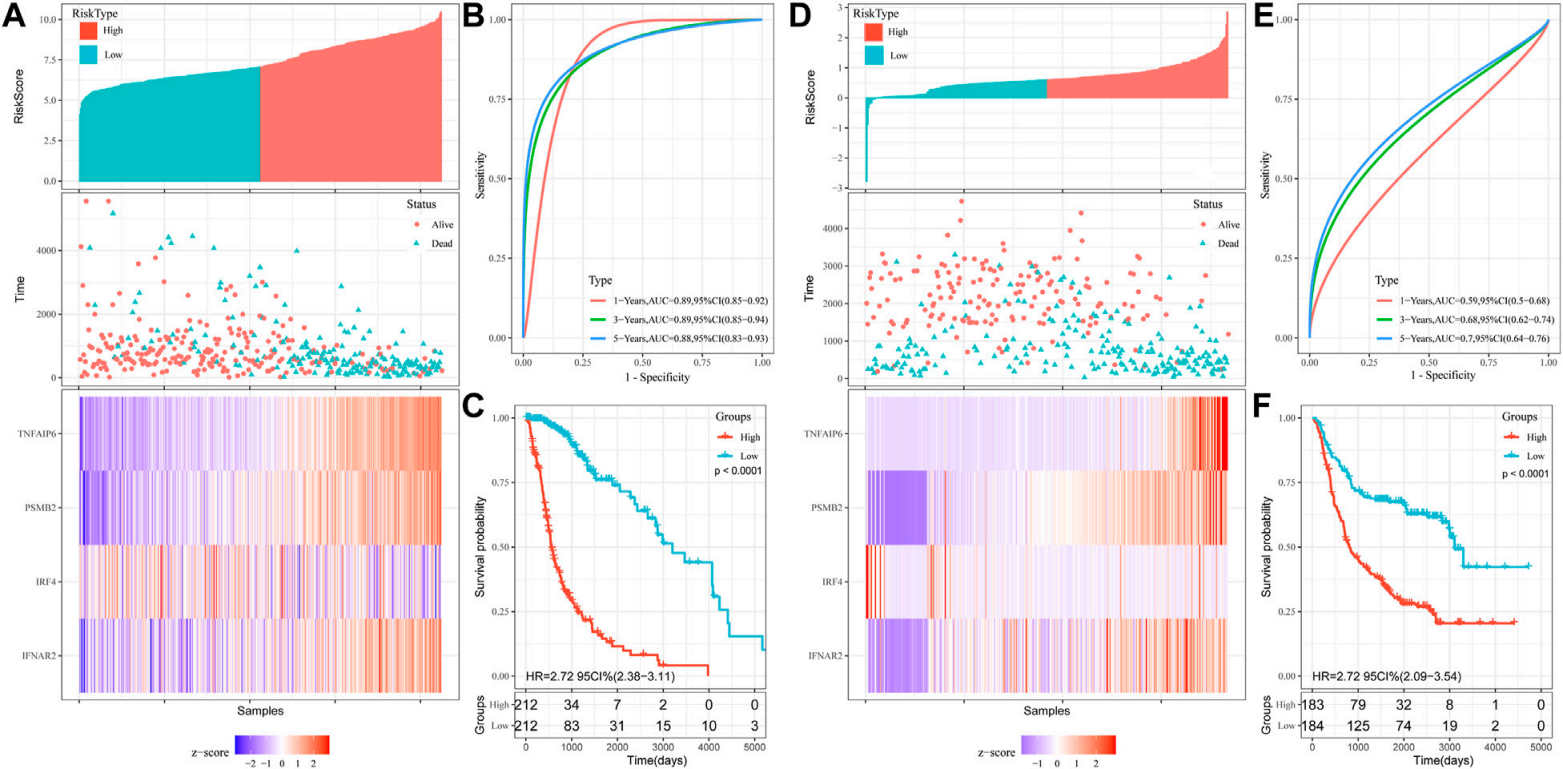
Construction of OS prediction model based on 4 genes in the TCGA dataset: **(A)** Heat maps of four genes in the high and low risk score groups. **(B)** Time-dependent ROC curve for OS. **(C)** Kaplan-Meier analysis. Validation of OS prediction model based on 4 genes in CGGA dataset: **(D)** Heat maps of four genes in the high and low risk score groups. **(E)** Time‐dependent ROC curve for OS. **(F)** Kaplan-Meier analysis.

Based on the IFN-γ gene set in MSigDB, 198 IFN-γ -associated genes were selected. First, we performed LASSO regression analysis to identify the following 19 IFN-γ -associated genes, CASP4, PSMA2, SERPING1, KLRK1, SLC25A28, IFIT2, LY6E, TNFAIP6, ISG20, PSMB2, ITGB7, BANK1, IRF4, NFKBIA, IFNAR2, PIM1, TXNIP, IFITM3 and METTL7B (Figures 1D,E). Subsequently, univariate Cox analysis was performed on these 19 genes to search for genes associated with patient OS and prognosis. Eighteen IFN-γ related genes were selected (*p* < 0.05) (Figure 2A). Finally, multivariate Cox analysis revealed the four genes significantly associated with patient prognosis (*p* < 0.05), namely IFNAR2, IRF4, PSMB2 and TNFAIP6 (Figure 2B), that were subsequently used to establish a risk assessment model. In order to determine the expression of these four genes in glioma. We performed qRT-PCR analysis and found that the expressions of TNFAIP6 (Figure 2C), PSMB2 (Figure 2D), IRF4 (Figure 2E) and IFNAR2 (Figure 2F) were significantly upregulated in glioma tissues (n = 30) compared to normal brain tissues (n = 10). The equation used for calculating risk assessment was as follows: Risk score = (0.4007 * IFNAR2 expression value) + (−0.0693 * IRF4 expression value) + (0.8667 * PSMB2 expression value) + (0.3424 * TNFAIP6 expression value). We considered the median score as the critical value and divided the samples into the high- and the low-risk groups. Our results showed that the survival of patients in the high-risk group was worse than that in the low-risk group. The expression profiles of these four genes in the two groups is illustrated as a heat map (Figure 3A). Based on these findings, we inferred that our risk model may be an efficient tool to predict glioma patient prognosis.

### The risk score model based on IFN-γ related genes could independently predict prognosis of glioma patients

Univariate COX regression analysis showed that risk score was significantly associated with prognosis (HR = 6.616, 95% CI = 4.701–9.313, *p* < 0.001; Table 2). Multivariate Cox regression analysis, after adjustment for other clinical characteristics, confirmed that risk score was independent of the clinical parameters (HR = 2.037, 95%CI = 1.236–3.357, *p* = 0.005) (Table 2). Kaplan-Meier analysis of differential survival between the two groups found that patients in the high-risk group had significantly worse survival (shorter survival duration and lower survival) than those in the low-risk group (*p* < 0.0001) (Figure 3C). To verify the superiority of our risk scoring model, relevant ROC curves were drawn. The AUC values of 1-, 3- and 5-year regions were 0.89, 0.89, and 0.88, respectively, (all AUC values >0.7; Figure 3B), indicating that the risk model had good predictive value.

**TABLE 2:** Univariate and multivariate analysis of the risk scores in TCGA database and CGGA database.

Datasets		Univariate		Multivariate	
	Variable	HR (95% CI)	*p*	HR (95% CI)	*p*
TCGA	Age	4.592 (3.352–6.291)	<0.001	1.252 (0.861–1.819)	0.240
	Gender	1.337 (0.998–1.791)	0.051	1.264 (0.913–1.751)	0.158
	Histology	8.802 (6.367–12.168)	<0.001	2.220 (1.454–3.390)	<0.001
	Karnofsky Score	0.489 (0.348–0.686)	<0.001	0.778 (0.533–1.137)	0.195
	Idh1 Status	0.111 (0.079–0.154)	<0.001	0.317 (0.188–0.534)	<0.001
	Risk Score	6.616 (4.701–9.313)	<0.001	2.037 (1.236–3.357)	0.005
CGGA	Age	2.897 (2.075–4.044)	<0.001	1.151 (0.797–1.662)	0.454
	Gender	1.131 (0.851–1.503)	0.396	0.996 (0.741–1.338)	0.978
	Histology	5.063 (3.787–6.768)	<0.001	2.537 (1.769–3.640)	<0.001
	Idh1 Status	0.226 (0.168–0.305)	<0.001	0.394 (0.277–0.562)	<0.001
	Risk Score	2.484 (1.855–3.327)	<0.001	1.585 (1.159–2.168)	0.004

### Validation of the risk scoring model

We used the CGGA database as a validation set to evaluate the reliability of our risk scoring model for IFN-γ -associated genes. The median score was taken as the critical value, and the samples were divided into the high- and low-risk groups. Patient OS status was assessed and the heat maps depicting the expressions of the selected four genes in the two groups were drawn (Figure 3D). Survival was consistently low in the high-risk group (Figure 3F). ROC curve analysis of validation sets assessed the prognostic efficiency of risk scoring model. The AUC value for 1-, 3-, and 5-year was 0.59, 0.68, 0.70, respectively (Figure 3E). In addition, univariate COX regression analysis showed that risk score significantly correlated with prognosis (HR = 2.484, 95%CI = 1.855–3.327, *p* < 0.001) (Table 2). Multivariate Cox regression analysis showed that risk score could independently predict prognosis (HR = 1.585, 95%CI = 1.159–2.168, *p* = 0.004) (Table 2). Collectively, these results suggest that risk score based on the selected four genes could efficiently predict patient prognosis.

### Correlation between risk scoring model, disease progression, and the nomogram

**FIGURE 4:**
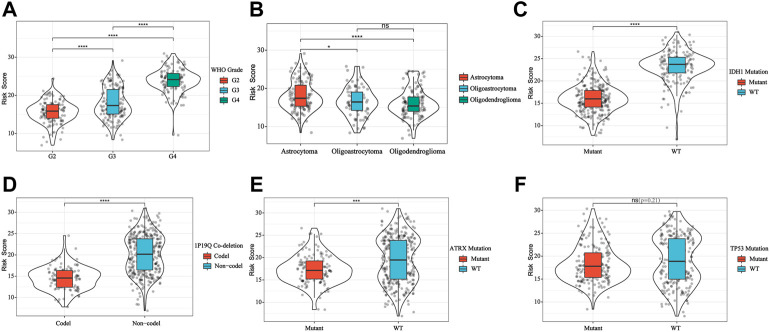
IFN-γ -associated gene scoring model was correlated with WHO Grade **(A)**, glioma subtype **(B)**, IDH1 **(C)**, 1P19Q co-deletion **(D)**, ATRX **(E)** and TP53 **(F)**. **p < 0.01, ****p < 0 .0001.

**FIGURE 5:**
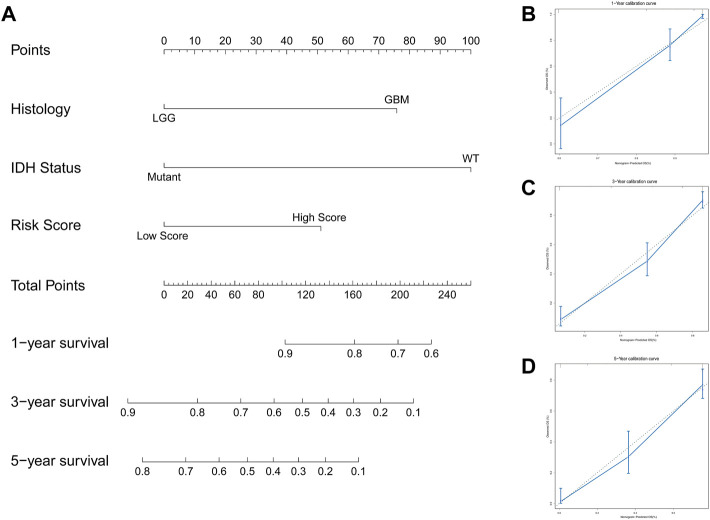
Constructed a nomogram based on the risk scoring model and independent clinical factors. **(A)** Nomogram predicts 1 -, 3 -, and 5-year OS for glioma patients. **(B–D)** Calibration curves for 1-, 3-, and 5-year OS.

We explored the relationship between the IFN-γ -associated gene scoring model and various clinicopathological factors, and found that patients with advanced tumor grade, Astrocytoma, wild-type IDH1, 1P/19Q non-co-deletion, and wild-type ATRX had significantly higher risk scores (*p* < 0.05, Figures 4A–E). The IFN-γ related gene score model was statistically correlated with a variety of clinicopathological factors; the higher the risk score, the worse the clinicopathological status. In addition, we constructed a nomogram based on the risk scores and independent clinical factors (age, sex, and tumor grade) (Figure 5A). The nomogram was used to predict OS rates at 1, 3, and 5 years. Calibration curves for 1-, 3-, and 5-year OS showed that the nomogram had good predictive accuracy for the TCGA dataset (Figures 5B–D).

### IFN-γ -associated gene scoring model predicts tumor microenvironment changes in glioma patients

**FIGURE 6:**
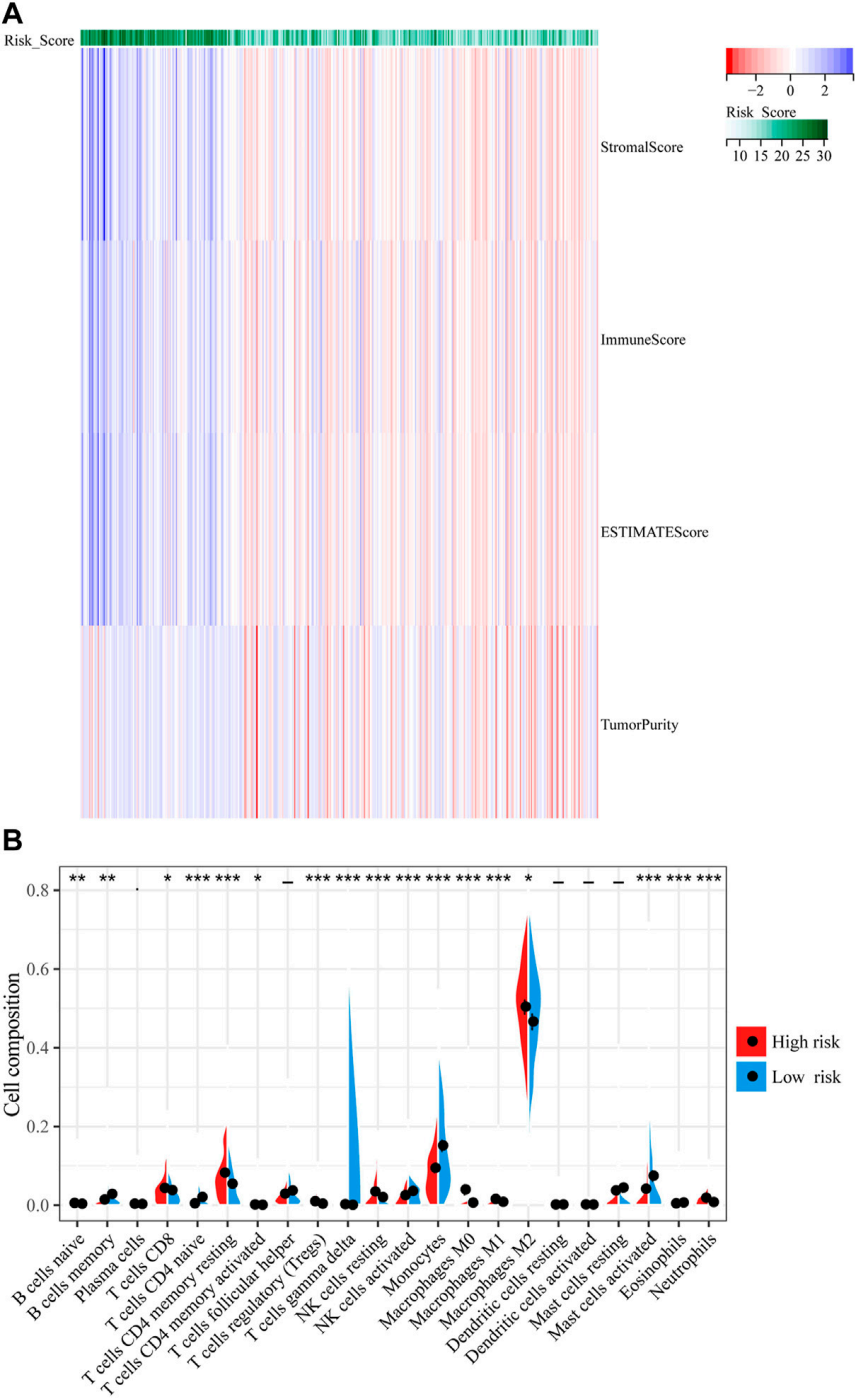
Correlation between IFN-γ -associated gene scoring model and tumor microenvironment. **(A)** The ESTIMATE algorithm to explore the relationship between the risk models and immune cell infiltration. **(B)** The percentages of 22 immune cells were assessed using CIBERSORT. **p < 0.01, ****p < 0 .0001.

To explore the association between immune response and IFN-γ related genes in gliomas whose data was obtained from TCGA, we applied the ESTIMATE algorithm to explore the relationship between the risk models and immune cell infiltration, where the immune score was positively correlated with risk score (Figure 6A). Next, we assessed the relative proportions of 22 types of infiltrating immune cells using the CIBERSORT. As shown in Figure 6B, CD8 T-cell, resting memory CD4 T-cell, monocytes, M2 macrophages, and activated mast cells had higher proportions among infiltrated immune cells. Furthermore, memory B-cell, naive CD4 T-cell, activated NK cells, and monocytes were negatively correlated with risk score, whereas CD8 T-cell, resting CD4 memory T-cell, regulatory T-cell (Tregs), M2 macrophages, resting NK cells, activated mast cells, and neutrophils were positively correlated with risk score (*p* < 0.05, Figure 6B).

**FIGURE 7:**
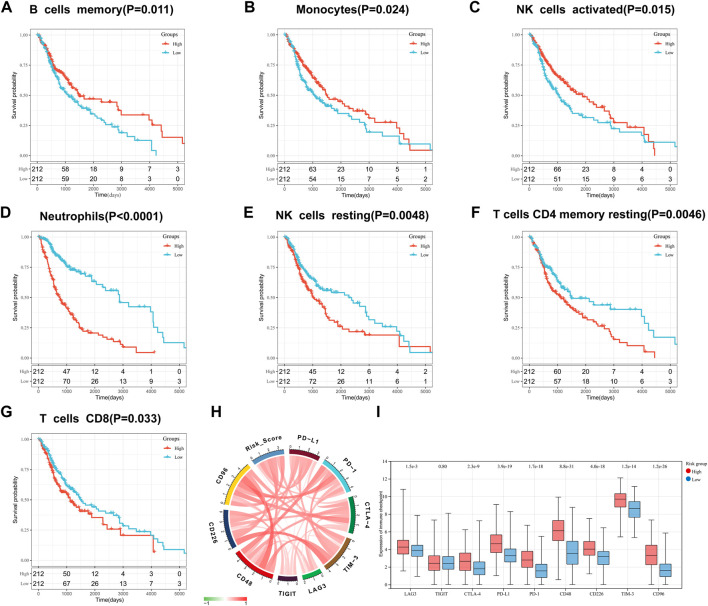
Prognostic value of infiltrating immune cells: memory B-cell **(A)**, monocytes **(B)**, activated NK cells **(C)**, neutrophils **(D)**, resting NK cells **(E)**, resting CD4 memory T-cell **(F)**, and CD8 T-cell **(G)**. Correlation between IFN-γ -associated gene scoring model and immune checkpoints: **(H)** Correlation Circos plots. **(I)** Expression of immune checkpoints in the high and low risk scoring group.

We also evaluated the prognostic value of 22 types of infiltrating immune cells and found that cells including memory B-cell (Figure 7A), monocytes (Figure 7B), neutrophils (Figure 7D), activated NK cells (Figure 7C), resting NK cells (Figure 7E), resting CD4 memory T-cell (Figure 7F), and CD8 T-cell (Figure 7G) was significantly associated with OS (*p* < 0.05). Neutrophils, resting NK cells, resting memory CD4 T-cell, CD8 T-cell higher dilatancy abundance was associated with poor OS, whereas a higher abundance of memory B-cell, monocytes, neutrophils, and activated NK cells indicated better OS. In summary, risk score statistically correlated with the altered proportion of most immune cells, suggesting that our IFN-γ-associated gene risk score model can predict the immunological status of glioma microenvironment.

### Correlation of interferon gamma gene risk assessment models with immune checkpoints

The association of our risk score with important checkpoint molecules, including PD-1, PD-L1, CTLA-4, LAG-3, TIM-3, TIGIT, CD226, CD48 and CD96, was evaluated in the TCGA dataset (Figure 7H). The correlation coefficient R between risk score and genes encoding immune checkpoint molecules is shown in supplementary Table 1. In addition, we found that the expression of LAG-3, CTLA-4, PD-L1, PD-1, CD48, CD226, TIM-3, and CD96 was significantly higher in high-risk groups than in low-risk groups (*p* < 0.0001; Figure 7 I).

## Discussion

Glioma is the most common primary malignant tumor of central nervous system. Conventional treatment modalities for glioma include surgery and radio- and chemo-therapy. However, none of these significantly improve the prognosis of glioma patients. Studies have shown that immunotherapy is a powerful strategy for clinical management of various cancers including glioma (Sanmamed and Chen, 2018; Riley et al., 2019; Daubon et al., 2020). Therefore, it is necessary to identify potential biomarkers to predict the survival in glioma patients. Moreover, immune-related biomarkers of TME will help to predict the patient immune response. To maximize the clinical benefits of glioma immunotherapy and improving patient prognosis, it may be a useful strategy to enrich the immune cell populations relevant for immunotherapeutic outcomes.

In tumors, IFN-γ acts as an effective apoptosis-inducing factor by directly inducing caspase-1 and caspase-8 in tumor cells (Chin et al., 1997). IFN-γ also plays an important role in regulating immune response; antigen presentation (Ivashkiv, 2018); inflammation; chemotactic signaling (Mauldin et al., 2016); modulating extracellular matrix, thereby affecting metastasis and tumor structure (Glasner et al., 2018); and activation and polarization of white blood cells (Burke and Young, 2019). Furthermore, IFN-γ plays an important role in inducing PD-L1 expression in glioma (Qian et al., 2018). So far, studies have focused on the role of IFN-γ in cancer progression and treatment; however, only a handful studies have investigated the role of IFN-γ-associated genes in glioma prognosis.

Here, we first selected 198 IFN-γ -associated genes. Among these, four genes (*IFNAR2, IRF4, PSMB2* and *TNFAIP6*) were identified as potential prognostic markers by univariate Cox analysis and LASSO regression analysis, and were used to construct prognostic models. Silginer et al. reported that silencing the gene encoding IFN alpha/beta receptor 2 (*IFNAR2*) leads to decreased expression of PD-L1 and major histocompatibility complex (MHC) proteins, thereby facilitating immune evasion of glioma cells (Silginer et al., 2017). Lei et al. reported that *IRF4* mRNA overexpression is associated with advanced pathological tumor grade and worse prognosis of glioma patients (Lei et al., 2021). Tan et al. reported that *PSMB2* knockdown inhibits HCC proliferation, invasion, and tumorigenesis (Tan et al., 2018), whereas Niu et al. reported that *TNFAIP6* is involved in inflammatory and immune response pathways (Niu et al., 2021). In our study, these four genes were involved in tumor development, inflammation, and immune response pathways. We used CGGA database to verify the validity and stability of our model. We observed significantly lower survival in the high-risk group than in the low-risk group (*p* < 0.0001). Univariate and multivariate Cox regression analysis confirmed that our model was an independent tool to predict patient prognosis (*p* < 0.05). Finally, a nomogram was established to validate the good performance of our risk model for predicting patient prognosis.

TME is a key factor regulating the development of malignant tumors (Quail and Joyce, 2017). Comprehensive understanding of the glioma TME will greatly improve the efficacy of glioma treatment strategies and prognosis of glioma patients. Our risk scoring model positively correlated with the immune score and matrix score of TME, indicating that the model was stable and accurate. Tumor-infiltrating immune cells are an important component of several cancers (Niu et al., 2020). CIBERSORT was used to evaluate the relative proportion of 22 types of infiltrating immune cells. M2 macrophages are the most important immune cell type and are involved in immunosuppression and tumor growth promotion (Zhu et al., 2017). Our results showed that M2 macrophages were more prevalent in the high-risk group. CD8 T-cell have the potential to treat glioblastoma *via* CAR T-cell therapy (Murphy and Griffith, 2016). We found that CD8 T-cell was significantly associated with OS (*p* < 0.05). Therefore, our risk score may correctly predict the status of glioma TME as well as patient outcomes.

Checkpoint inhibitors are playing an increasingly important role in glioma immunotherapy (Ghouzlani et al., 2021). Among them, PD-1 and its ligand PD-L1 significantly modulate immunotherapy outcomes in various tumors (Wang et al., 2019). The binding of PD-1 to PD-L1 facilitates cancer immune evasion *via* inhibiting T-cell function (Ricklefs et al., 2018). In this study, we observed a correlation between the risk scoring model and PD-L1 using the TCGA dataset (R = 0.54). The mechanism of positive correlation between the risk score and PD-L1 in glioma may be related to IFN/PD-L1 axis of anti-PD-1/PD-L1 treatment (Qian et al., 2018). Biomarkers identified based on risk score model can accurately predict the efficacy of PD-L1 inhibitor therapy, thereby allowing glioma patients to benefit more from PD-L1 blocker therapy in the future. Cytotoxic T-lymphocyte associated protein 4 (CTLA-4) can affect the treatment of advanced cancer and targeting drugs have been used for treating different types of cancer (Rotte, 2019). CTLA-4 overexpression in glioma TME can induce immune cell infiltration (Liu et al., 2020). Our risk score model positively correlated with CTLA-4 (R = 0.33), which may be associated with CTLA-4 blocking and increased number of IFN-γ -producing tumor-infiltrating T-cell (Giles et al., 2018). Collectively, these results suggest that our risk score model can predict patient prognosis as well as response to immune checkpoint therapy.

We generated a risk model based on four IFN-γ -associated gene, which were selected based on rigorous screening criteria. The specificity and reliability of the model were verified in CGGA data sets. In addition, we generated a nomogram based on the clinical characteristics of patients. Further explore the correlation among TME, infiltrating immune cells, and immune checkpoint inhibitors, which may be useful in the future for effectively predicting prognosis of glioma patients in clinical settings. However, our study has limitations. Since our study mainly involved *in silico* analysis of mined data, the results should be validated in laboratories and clinics using a larger number of glioma patients in the future.

In conclusion, we constructed a risk score model based on IFN-γ-related genes that are closely related to the immune status of TME. This model can better predict the prognosis of glioma patients and help in optimizing glioma immunotherapy.

## Data Availability

The original contributions presented in the study are included in the article/Supplementary Material, further inquiries can be directed to the corresponding author.
