# Supplementary material for: Interferon gamma-related gene signature based on anti-tumor immunity predicts glioma patient prognosis
Source: Front Genet. 2023 Jan 13;13:1053263. doi: 10.3389/fgene.2022.1053263 (PMC9880184; doi:10.3389/fgene.2022.1053263)
Supplement: Supplementary file 1 [file Table1.DOCX]

Supplementary Material

# Supplementary Tables

**Supplementary TABLE 1 The R values between Risk Score and immune checkpoint genes**

| Immune Checkpoint | Risk Score |
| --- | --- |
| LAG3 | 0.123976 |
| TIGIT | 0.031436 |
| CTLA-4 | 0.330978 |
| PD-L1 | 0.542031 |
| PD-1 | 0.442659 |
| CD48 | 0.561542 |
| CD226 | 0.520939 |
| TIM-3 | 0.43201 |
| CD96 | 0.566293 |
