# Supplementary material for: Interferon gamma-related gene signature based on anti-tumor immunity predicts glioma patient prognosis
Source: Front Genet. 2023 Jan 13;13:1053263. doi: 10.3389/fgene.2022.1053263 (PMC9880184; doi:10.3389/fgene.2022.1053263)
Supplement: Supplementary file 2 [file Table2.DOCX]

CGGA original data：https://www.jianguoyun.com/p/DV6t_pEQlIL8ChjfltoEIAA

Clinical features original data：https://www.jianguoyun.com/p/DVu2NnMQlIL8ChjlltoEIAA

Immune checkpoints original data：https://www.jianguoyun.com/p/DXx028wQlIL8ChjnltoEIAA

pcr original data：https://www.jianguoyun.com/p/DXT526oQlIL8ChjrltoEIAA

TCGA original data：https://www.jianguoyun.com/p/DSzaa_4QlIL8ChjsltoEIAA

Univariate and multivariate analysis：https://www.jianguoyun.com/p/DbVCo4IQlIL8ChjultoEIAA

R code: https://www.jianguoyun.com/p/DdHWMpUQlIL8ChifvNoEIAA
